# The complete mitochondrial genome of *antheraea pernyi* strain qing_6 (lepidoptera: Saturniidae)

**DOI:** 10.1080/23802359.2020.1823903

**Published:** 2020-09-18

**Authors:** Yun-Can Liu, Xin-Yu Li, Dong-Bin Chen, Yan-Qun Liu, Xi-Sheng Li

**Affiliations:** a Department of Sericulture, College of Bioscience and Biotechnology, Shenyang Agricultural University, Shenyang, China; b Sericultural Institute of Liaoning Province, Fengcheng, China

**Keywords:** *Antheraea pernyi*, Qing_6, mitochondrial genome, genetic variation

## Abstract

Here, we report the complete mitochondrial (mt) genome of *Antheraea pernyi* Qing_6, an improved strain serving silk production and edible insect resource for 50 years in Northeast China. The circular mt genome spans 15,572 bp in length and contains 37 typical coding genes (13 protein-coding genes, two ribosomal RNA genes, 22 transfer RNA genes). Its A + T-rich region is 552 bp in size exhibiting identical sequence with the first modern improved strain Qinghuang_1. Comparison analysis identified only 12 variable sites (5 substitutions and 7 indels) between Qing_6 and Qinghuang_1. The phylogenetic analysis also clustered Qing_6 and Qinghuang_1 together first, which was in line with the breeding history of the two strains.

Chinese oak silkmoth, *Antheraea pernyi* is one of traditional edible and silk-producing insect resources in China, and the detailed record of the artificial rearing of this insect was found in 1651 (Li et al. 2017). This insect has been considered the edible insect with the highest potential (Ambühl 2019). To develop oak silkmoth industry, more than 100 strains of this insect have been developed with diverse agronomic characters. Like Qinghuang_1, the first modern improved strain of *A. pernyi*, Qing_6 is one such strain developed by the Sericultural Institute of Liaoning Province in 1956 through a systematic selection method from a local population ‘Aiyang’ from Liaoning Province. As one of the excellent strains, Qing_6 have served silk production and edible insect resource for 50 years in Northeast China. In the present study, the complete mitochondrial (mt) genome of this strain was determined for the first time, providing the basic genetic information for future improvement.

The inbred strain Qing_6 has been successively preserved by the Sericultural Institute of Liaoning Province (N40°28′; E123°59′), Fengcheng, China since 1956. When eggs hatched, the first-instar larvae were immediately preserved in 95% ethanol. The specimens were stored with the collection number of OAK_SILKWORM_QING_6 at Department of Sericulture, Shenyang Agricultural University, China. A single larva was used to extract the total genomic DNA. Two over-lapping long fragments were PCR amplified with specific primer pairs . PCR products were purified and sequenced on the Illumina PE 150 platform, and sequence assembly was performed with the method of ABySS and Shovill provided on the web server https://usegalaxy.eu/. The mt genome of Qing_6 has been deposited in GenBank under accession no. MT890592.

The full mt genome of Qing_6 is 15,572 bp in length, and contains a typical mt genomic complement including 13 protein-coding genes (PCGs), 22 tRNA genes, two ribosomal RNA genes and a non-coding A + T-rich region, exhibiting an identical genomic component and gene order with known Bombycidea species. The A + T-rich region is 552 bp in size showing an identical sequence with the first modern improved strain Qinghuang_1. Sequence comparison analysis identified only 12 variable sites (5 substitutions and 7 indels) between Qing_6 and Qinghuang_1 across the whole mt genome, while 81 variable sites (76 substitutions and 5 indels) between Qing_6 and Yuzao_1 (Liu et al. 2008).

To assess the intraspecific phylogenetic relationships of *A. pernyi* samples, the phylogenetic relationship (Figure 1) based on the nucleotide sequences of 14 whole mt genomes, including six *A. pernyi* strains available to date (Liu et al. 2008; Zhang and Wu 2016; Zhang et al. 2016; Zhao et al. 2016), three *Antheraea* species, five closely related Saturniidae species, was built using Bayesian inference method with GTR + G + I model in Mrbayes 3.1.2 (Huelsenbeck and Ronquist 2001). In the phylogenetic tree obtained, the posterior probabilities were high for all nodes. Bayesian inference analysis recovered two groups for the six *A. pernyi* strains used, one contained Qing_6 and Qinghuang_1 and the other contained four strains (Yuzao_1, Yu_6, Yu_7 and 731). According to the breeding history, Qing_6 and Qinghuang_1 were derived from ‘Aiyang’ population of Liaoning Province, and the other four strains from ‘Lushan’ population of Henan Province. The mt genome-based phylogenetic analysis clustered Qing_6 and Qinghuang_1 together first, which was in line with their breeding histories.

**Figure 1.**
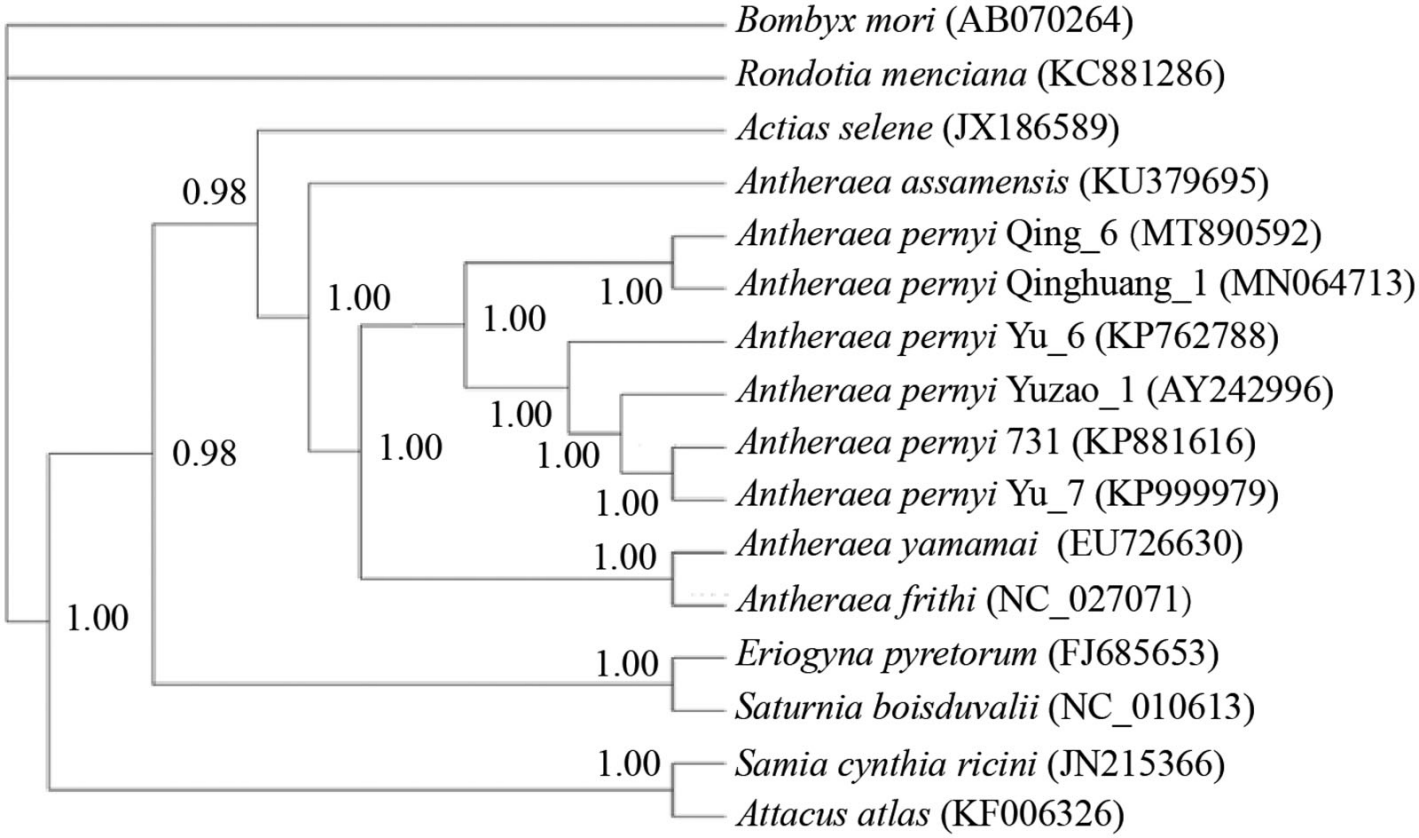
Phylogenetic tree inferred from the whole mt genome sequences using bayesian inference method with GTR + G + I model. The posterior probability values are indicated at the nodes. GenBank accession numbers are listed following the name of each species or strain.

## Data Availability

The data that support the findings of this study are openly available in GenBank of NCBI at https://www.ncbi.nlm.nih.gov, reference number MT890592.
